# Taper Preparation Variability Compared to Current Taper Standards Using Computed Tomography

**DOI:** 10.1155/2012/265695

**Published:** 2012-02-29

**Authors:** Richard Gergi, Joe Abou Rjeily, Nada Osta, Joseph Sader, Alfred Naaman

**Affiliations:** ^1^Department of Endodontics, Faculty of Dentistry, Saint-Joseph University, P.O. Box 166255, Beirut, Lebanon; ^2^Department of Prosthodontics, Faculty of Dentistry, Saint-Joseph University, P.O. Box 166255, Beirut, Lebanon

## Abstract

*Introduction*. The purpose of this study was to compare the taper variation in root canal preparations among Twisted Files and PathFiles-ProTaper .08 tapered rotary files to current standards. *Methods*. 60 root canals with severe angle of curvature (between 25° and 35°) and short radius (*r* < 10 mm) were selected. The canals were divided randomly into two groups of 30 each. After preparation with Twisted Files and PathFiles-ProTaper to size 25 taper .08, the diameter was measured using computed tomography (CT) at 1, 3, and 16 mm. Canal taper preparation was calculated at the apical third and at the middle-cervical third. *Results*. Of the 2 file systems, both fell within the ±.05 taper variability. All preparations demonstrated variability when compared to the nominal taper .08. In the apical third, mean taper was significantly different between TF and PathFiles-ProTaper (*P* value < 0.0001; independent *t*-test). Mean Taper was significantly higher with PathFile-ProTaper. In the middle-cervical third, mean Taper was significantly higher with TF (*P* value = 0.015; independent *t*-test). *Conclusion*. Taper preparations of the investigated size 25 taper .08 were favorable but different from the nominal taper.

## 1. Introduction

Cleaning and shaping the root canal has been recognized as an important phase in endodontic therapy [1, 2]. When the root canal system (RCS) is cleaned and shaped to a specific size, the goal is to achieve an adequate seal particularly at the apex and coronal aspect in order to prevent leakage [3]. This is best accomplished when the file taper reported by the manufacturer is accurate and the taper of the canal following instrumentation corresponds to the taper of the file. Variation in file taper can affect the quality of endodontic obturation, which affects the overall success of the health and longevity of the tooth [4]. Variations in taper can also lead to unnecessary frustration by the clinician during obturation if the advertised file taper does not correlate as a result of manufacturing error. Input from Ingle [5], Heuer [6], and others led to the development of international standards on size, taper, and performance of endodontic files [7]. Revisions were made to the International Standards Organization (ISO) leading to the specification 101 of the American National Standards Institute/American Dental Association (ANSI/ADA). This specification states the taper dimensional requirements for endodontic files of any taper [8]. Thus the ISO 3630-1 and the ANSI/ADA specification 101 currently serve as the standard to which endodontic file is compared.

 Despite the most advanced technology in manufacturing of dental instruments, variations in endodontic file tapers still exist [9]. A recent study comparing the taper variability among  .06 tapered rotary nickel titanium (NiTi) files found that of all the files brands evaluated demonstrated taper variability [10]. According to the ANSI/ADA specification 101, the allowable taper variation tolerance, for any size file or root canal preparation (RCT), is ±0.05 [8]. This means that if a manufacturer states that the nominal file taper is  .08, the taper can vary between  .03 and  .13 and still fall within the current acceptable standards on taper; a large amount of variance might occur and still be within the standard.

 To date, very few studies have been conducted analyzing root canal preparation taper variability with NiTi rotary endodontic files to current standards. The purpose of this study was to compare the variability among the Twisted File (TF, SybronEndo, Orange, CA) and the PathFile-ProTaper system (Denstply, Maillefer, Ballaigues, Switzerland) of size 25,  .08 tapered NiTi rotary files.

## 2. Materials and Methods

In total 60 root canals with completely formed apices and severe angles of curvature 25° < *α* < 35° [11] and short radii <10 mm [12] stored in 10% buffered formalin were selected for the present study. Access cavity was prepared using a 4 high-speed round carbide bur (Dentsply, Maillefer) with water spray. A size 10 K-file (Dentsply, Maillefer) was placed into the canal until it was visible at the apical foramen and the working length established 0.5 mm short of this length. If the apical diameter was larger than a 10 K-file, the tooth was excluded from the study and another tooth having a severe angle of curvature and short radii was selected. For more uniform samples, the crowns were flattened with steel discs and a final dimension of 18 mm working length was achieved for each tooth.

Roots were embedded into transparent acrylic (Orthoplast; Vertex, Zeist, The Netherlands). The teeth were randomly divided into two experimental groups. Root canals were instrumented by the same operator using a standardized technique. All root canals were instrumented to the working length with sizes 10 and 15 K-files using a step-back technique. Canals that were larger than ISO size 15 were discarded.

Group 1 of 30 teeth was prepared using Twisted File instruments developed by SybronEndo according to the manufacturer's recommendations.

1. The shaping procedure commenced with TF size 25 and  .08 taper. The coronal 1/3 or 2/3 of the root canal was shaped if passive penetration was possible.
2. TF size 25 and  .06 taper was inserted and used until 2 mm short of working length (WL).
3. Shaping continued with  .04 taper size 25 instrument to the WL.
4. TF size 25,  .06 taper was taken to WL.
5. A .08 taper size 25 instrument was taken to WL.

Group 2 of 30 teeth was prepared using the PathFile and ProTaper files according to the manufacturer's recommendations.

1. The shaping procedure commenced with PathFile 1 (.02 taper size 13), followed by 2 (.02 taper size 16), and then by 3 (.02 taper size 19) to WL.
2. This was followed by the use of ProTaper S1 then S2 to WL.
3. Shaping continued with F1 finishing instrument (.07 taper size 20) followed by F2 (.08 taper size 25) to WL. S1 and S2 instruments were used with a brushing motion while nonbrushing motion was applied to F1, F2, and TF instruments.

Consequently the final apical preparation resulting was standardized to  .08 taper size 25 for both groups.

Each instrument was used with the 1 : 75 reduction rotary hand-piece (06 XE; Micro-Mega); the speed of rotation was maintained at 500 rpm for the TF and 350 for the PathFile-ProTaper files according to the manufacturer's recommendation. Canals were irrigated between instruments with 3 mL of a 5.25% NaOCl using a disposable syringe on which an Endo-Eze (Ultradent, South Jordan, USA) irrigator tip was mounted. Glyde (Dentsply, Maillefer) was used as a lubricant during instrumentation, and when root canal instrumentation was completed, 1 mL of 15% EDTA (Wizard, Rehber Kimya San., Istanbul, Turkey) was applied for 1 min and the canals flushed again with 3 mL of NaOCl.

**Figure 1:**
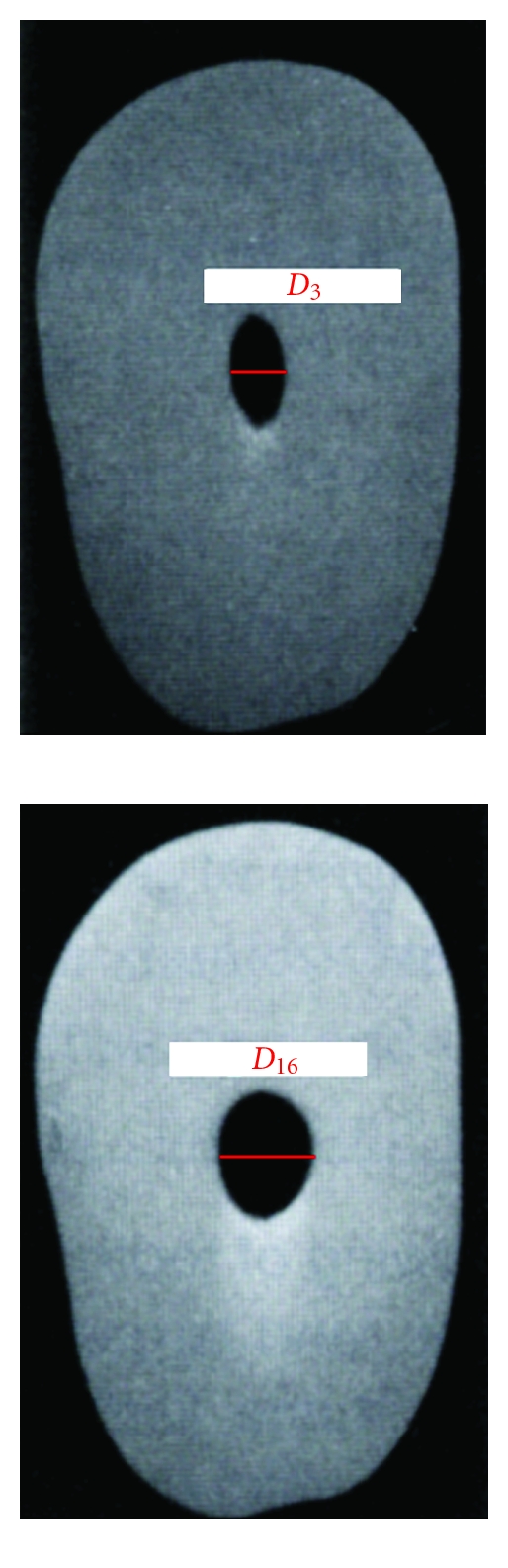
Diameter measure D3 and D16 after root canal preparation.

After root canal preparation, all teeth were scanned by spiral CT (Toshiba-002A; Toshiba, Tochigi-Ken, Japan). The sections were 1 mm thick from apical to the canal orifice. Three sections from each tooth, the number of the tooth, and its level were archived onto a magnetic optical disc (EDM 650B; Sony Corp., Tokyo, Japan). The first two sections were at 1 and 3 mm from the apical end of the root. The third section was at 16 mm from the apex. Taper was determined from the diameter at D_3_ and D_16_ (Figure 1) of each root canal preparation using the equation: Taper = D_16_ diameter − D_3_ Diameter (mm)/Distance between D_16_ and D_3_, where D_16_ and D_3_ are the shortest distance from the mesial edge to the distal edge of the instrumented canal. This equation was obtained from the ISO 3630-1 protocol for determining file taper with the measured diameter locations at D_16_ and D_3_.

According to the ProTaper manufacturer, the stated .08 file taper is accurate for the first 3 mm, with a variable taper beyond 3 mm. Because of the variable taper of the ProTaper file, another taper measure was evaluated in the first 3 mm of each file preparation for all groups using the equation: Taper = D_3_ diameter − D_1_ Diameter (mm)/Distance between D_3_ and D_1_. Based on the taper measurements, the percent difference from the nominal taper value was calculated for each file preparation at D_3_, D_1_ and D_16_, D_3_.

## 3. Results

The calculated taper file preparation at D_1 _ and D_3_ is summarized in Table 1. The 2 system preparations fell within the ANSI/ADA specification 101 for taper variability of ±.05. In the apical third, mean taper was significantly different between TF (7.30 ± 0.64) and PathFile-ProTaper (8.436 ± 0.75) (*P* value < 0.0001; independent *t*-test). Mean Taper was significantly higher with PathFile-ProTaper, and the magnitude of difference was significantly higher (Partiel Eta Squared = 0.708). Moreover, mean taper was significantly different from 8 percent (*P* value = 0.017; one sample *t*-test).

The calculated taper file preparation at D_16 _ and D_3 _ is summarized in Table 2. Mean taper was significantly different between TF (8.16023 ± 0.152618) and PathFiles-ProTaper (8.06508 ± 0.141301) (*P* value = 0.015; independent *t*-test). Mean Taper was significantly higher with TF. Moreover, mean taper was significantly different from 8 percent (*P* value = 0.017; one sample *t*-test). However, the 2 system preparations fell within the ANSI/ADA specification 101 for taper variability of ±.05.

The majority of the taper measurements were different than the nominal taper with Pathfiles-ProTaper preparations being larger than the TF in the apical third. In the rest of the root canal (middle third and cervical third), TF preparations were larger than the Pathfiles-ProTaper system.

## 4. Discussion

Root canal instrumentation with rotary NiTi files improves preparation quality, particularly in terms of reducing the occurrence of ledges, zips, and root canal transportation [13]. To investigate the efficiency of instruments and techniques developed for root canal preparation, a number of methods have been used to compare canal shape before and after preparation. One of these methods is radiography. Its advantage is that no physical intervention is required; however, it only provides a two-dimensional image and a cross-section of the root canal is impossible to observe [14, 15]. The “Serial Sectioning Technique” of Bramante et al. [16], is a commonly used method. This technique allows comparison between instrumented and uninstrumented canals but a complicated set-up is required and physical sectioning of the teeth before preparation can result in unknown tissue changes and loss of material [16]. CT imaging techniques have been evaluated as noninvasive methods for the analysis of canal geometry and efficiency of shaping techniques [17–20]. With this technique, it is possible to compare the anatomic structure of root canal after instrumentation.

The result of the current study indicates that both NiTi systems analyzed fell within the allowable taper variability preparation of ±.05 in accordance with ANSI/ADA specification 101 [8]. Despite the establishment of ISO and ANSI/ADA, there is still a large amount of variation within the standard regarding files taper preparation. The results indicate that both brands studied exhibited taper preparations that were generally different than nominal taper with the largest difference displayed in the apical third for both brands. Although there was statistical significance between taper preparations of both systems, the corresponding taper deviation is very small to be of clinical concern. Previous studies comparing other rotary NiTi brands demonstrated also taper variability [3, 10]. Zinelis et al. [21] reported that none of the files studied complied with nominal size but most were within the ISO limits of tolerance. Although the reported accuracy of the investigated size 25,  .08 taper endodontic instruments is favorable, future studies should include preparation measurements of  .08 taper files with diameter other than size 30. In addition accurately manufactured gutta-percha cones are also important to match the diameter and taper of the last instrument used. Thus, future studies could also include the correlation of endodontic instrument diameter/taper measurements with the associated measurements of same size gutta-percha cones.

## Figures and Tables

**Table 1:** Mean percent difference and standard deviation from  .08 nominal taper in the apical third among 2 systems.

D3-D1/2	Groups	*N*	Mean	Standard deviation
Taper	Twisted File	30	7.30383*	0.638077
Taper	PathFile-ProTaper	30	8.43600*	0.750844

*Significant at *P* value <0.0001.

**Table 2:** Mean percent difference and standard deviation from  .08 nominal taper in the middle-cervical third among 2 systems.

D16-D3/13	Groups	*N*	Mean	Standard deviation
Taper	Twisted File	30	8.16023*	0.152618
Taper	PathFile-ProTaper	30	8.06508*	0.141301

*Significant at *P* value =0.015.
